# Evaluation of hemolysis in patients supported with Impella 5.5: a single center experience

**DOI:** 10.1186/s13019-025-03352-7

**Published:** 2025-03-01

**Authors:** Jessica S. Clothier, Serge Kobsa, Lynette Lester, Nithya Rajeev, Markian Bojko, Jonathan Praeger, Mark Barr, Raymond Lee

**Affiliations:** Keck School of Medicine, Division of Cardiac Surgery, University of Southern California, 1520 San Pablo St Suite 4300, Los Angeles, CA 90033 USA

**Keywords:** Hemolysis, Percutaneous LVAD, Blood transfusions, Mechanical circulatory support, Impella 5.5

## Abstract

**Background:**

Hemolysis, variably defined in mechanical circulatory support (MCS), is understudied in percutaneous left ventricular assist devices. We characterize hemolytic sequelae of Impella 5.5-supported patients in the largest series to date.

**Methods:**

All Impella 5.5 patients at our center from 2020 to 2023 were identified (*n* = 169) and retrospectively reviewed. Patients with a plasma free hemoglobin (PfHb) recorded (and not previously elevated) were included (*n* = 123). The top (high hemolysis [HH], *n* = 26) and bottom (low hemolysis [LH], *n* = 25) quintiles were categorized based on PfHb levels. Analysis between groups identified factors associated with hemolysis.

**Results:**

HH patients had higher admission SCAI stages (*p* = 0.008), more Impella 5.5 days (23.5 v 10.0, *p* = 0.001), more additional MCS (16/26 [61.5%] v 6/25 [24.0%], *p* = 0.015), and more transfusions of packed red blood cells (12.5 v 4.0, *p* = 0.001), fresh frozen plasma (2.5 v 0.0, *p* = 0.033), and platelets (3.0 v 0.0, *p* = 0.002). Logistic regression identified additional MCS (OR 10.82, *p* = 0.004) and more Impella days (OR 1.13 *p* = 0.006) as hemolysis risk factors. Eleven (44%) LH and 19/26 (73%) HH patients died, with no significant differences between postoperative complications. Compared with those who died, HH survivors had fewer platelet transfusions (2.0 vs. 5.0, *p* = 0.01) and less PfHb elevation days (3.0 v 6.0, *p* = 0.007).

**Conclusions:**

Hemolysis in this high-risk cohort has a poor prognosis. HH patients spent more days on Impella 5.5, needed more MCS, and required more blood product transfusions.

**Supplementary Information:**

The online version contains supplementary material available at 10.1186/s13019-025-03352-7.

## Background

Hemolysis, defined as the lysis of circulating red blood cells (RBCs), is associated with mortality and poor outcomes [1, 2]. A low level of hemolysis exists by default in patients on mechanical circulatory support (MCS) when mechanical injury is incurred to RBCs [1], with causative factors comprised of shear stress, flow acceleration, and RBC contact with device surfaces [2]. There is no universally accepted algorithm or definition to establish clinically significant hemolysis in the setting of MCS [1]. Furthermore, hemolysis is particularly understudied in percutaneous left ventricular assist devices (LVADs).

Studies and guidelines have previously used various laboratory markers in attempts to define hemolysis, which include plasma free hemoglobin (PfHb), lactate dehydrogenase (LDH), haptoglobin, bilirubin, and hemoglobinuria. Interagency Registry for Mechanically Assisted Circulatory Support (INTERMACS) guidelines have suggested that PfHb > 20 and LDH > 2.5 times the normal value are indicative of hemolysis [1, 3]. Other studies have suggested that a slightly higher level of PfHb (> 40), with the addition of either LDH > 2.5 times normal, hemoglobinuria, or clinical signs such as anemia in the absence of active bleeding or renal failure would appropriately define hemolysis on MCS [4–8]. Of note, LDH, also an inflammatory marker, is known to be nonspecific and has been thought to be an unreliable definer of hemolysis [2, 9]. 

In recent years, certain characteristics that could contribute to hemolytic events in percutaneous devices have been recognized, for example pump malfunction or thrombosis, positional problems (triggering “suction alarms”), and insufficient preload [2, 3, 6, 10]. A consensus statement of the MCS academic research consortium published in 2020 categorized hemolytic events into “major” and “minor,” using some of these abovementioned mechanical characteristics but still employing the previous INTERMACS reported PfHb level > 20 or LDH > 2.5x normal as a qualifying criteria for an “adverse event.” [3].

One study, with hemolysis defined as PfHb > 40, aimed to examine the predictive value of these INTERMACS markers among cardiogenic shock patients receiving an Impella device (a percutaneous LVAD manufactured by Abiomed, Danvers, MA) and found that while an increase in delta PfHb was highly predictive of hemolysis, an increase in delta LDH was not [9]. Of note, US Food and Drug Administration (FDA)-approved study protocol definitions of hemolysis for percutaneous MCS devices have specifically required PfHb > 40 to be recorded at two different points in time [7, 8, 11–13]. 

Notably, medical device companies benchmark test products on an FDA-approved model that does not utilize PfHb as there is no way to incorporate a kidney into the testing circuit (Figure S1) [14, 15]. This model employs Modified Index of Hemolysis (MIH), which represents rate of blood damage over time, as an alternative (Figure S2) [14, 15]. This imperfection of the device approval model makes a theoretical PfHb “cut-off value” for hemolysis difficult to ascertain.

Despite poor consensus on the definition of clinically significant hemolysis in MCS, we aim to study this complication in a real-world, clinically relevant manner in a series of patients supported with the Impella 5.5 percutaneous LVAD (Abiomed; Danvers, MA), which, to our knowledge, has not yet been reported. In the largest series to date, we characterize hemolytic sequelae of Impella 5.5-supported patients.

## Methods

This study was approved by the Institutional Review Board of the University of Southern California (IRB # HS-23-00521).

### Study design and patients

**Fig. 1:**
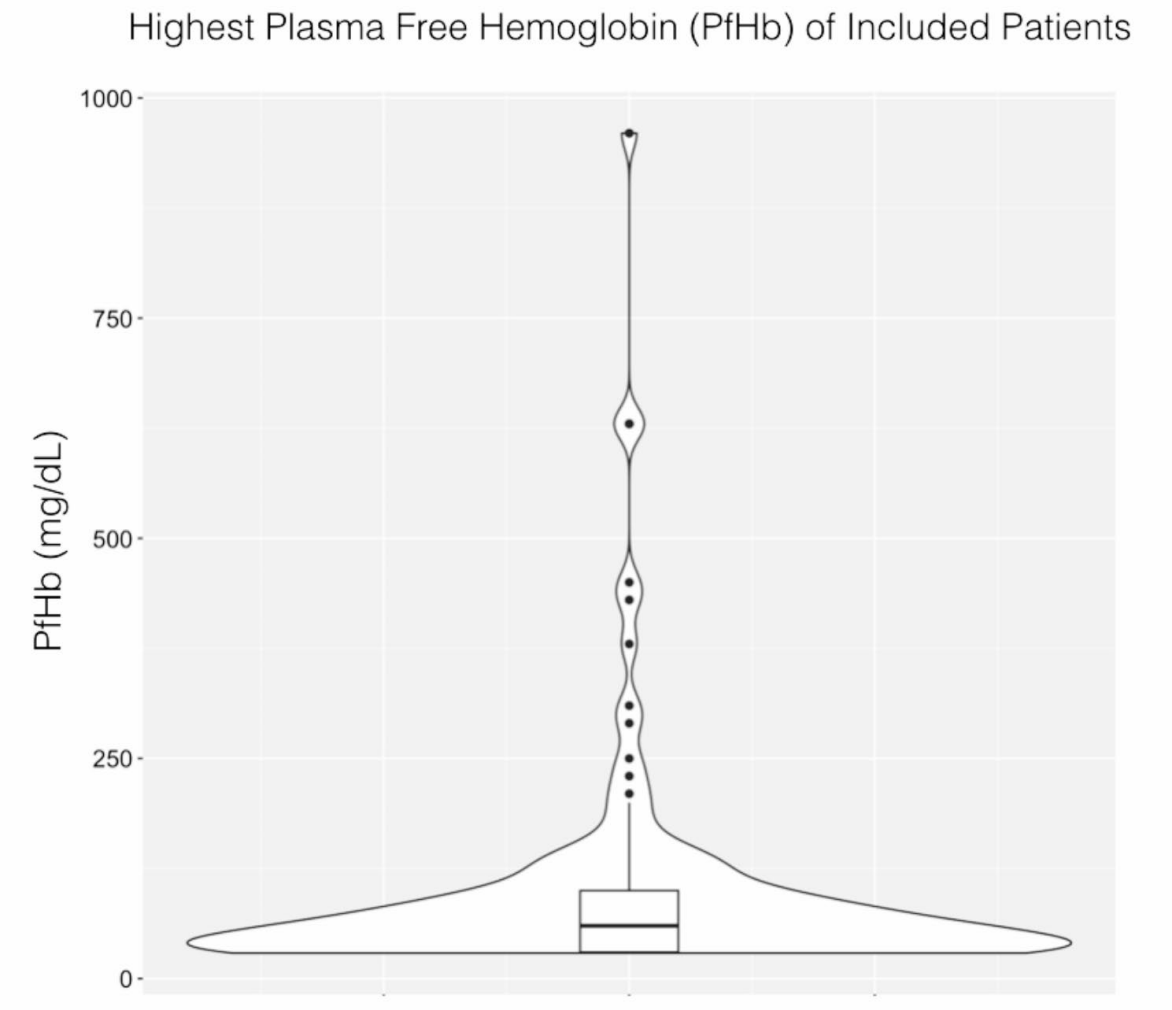
Violin plot depicting distribution of highest plasma free hemoglobin (PfHb) of included patients. The box plot within shows the median (60, solid middle line) and interquartile range (30–100, box ends) as well as outliers (black dots). For purposes of creating the figure, all values of “<30” were converted to 30

**Fig. 2:**
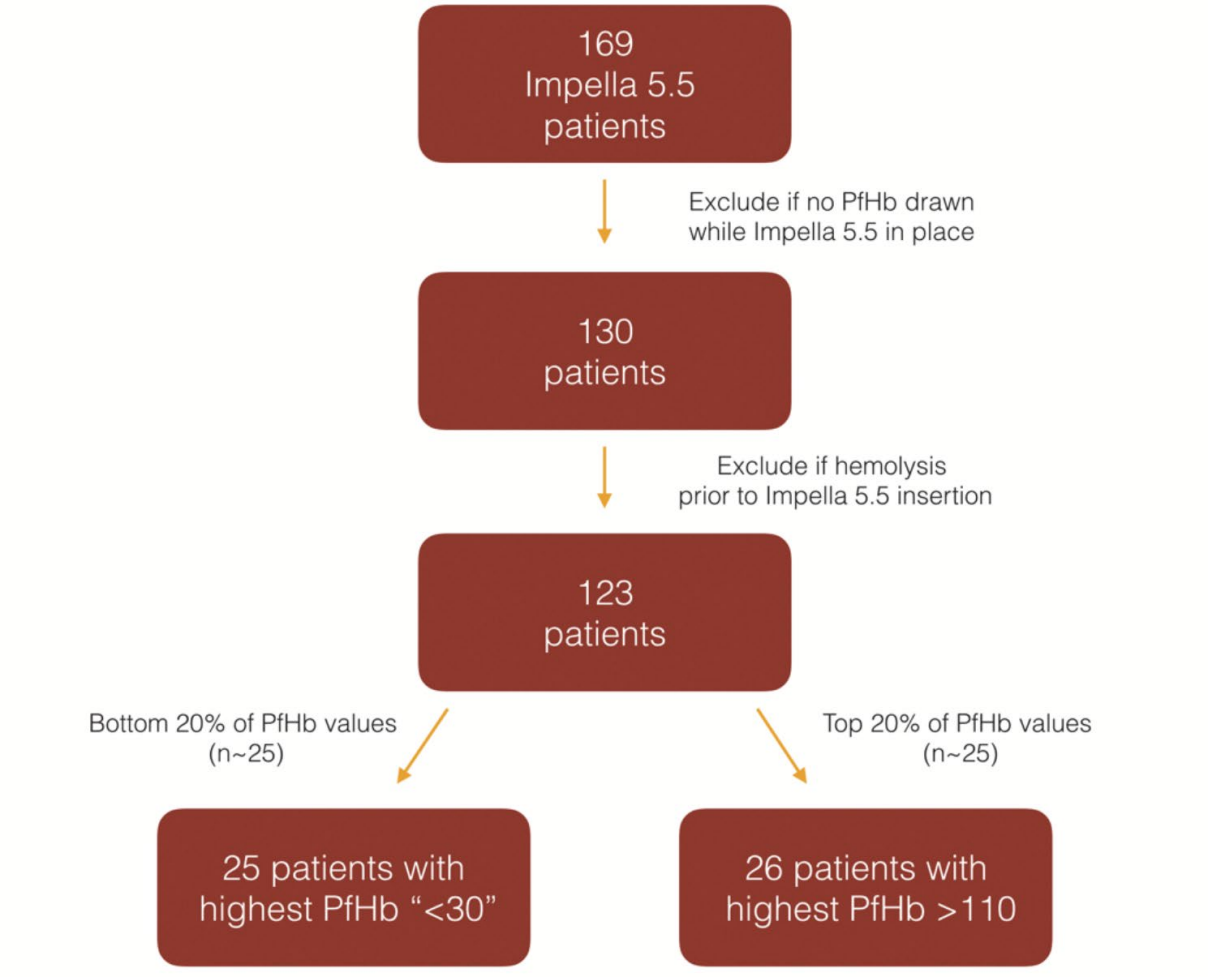
Allocation of high hemolysis (HH) and low hemolysis (LH) groups

All consecutive Impella 5.5 patients at our center from 2020 to 2023 were identified (*n* = 169) and retrospectively reviewed. Any patient with a PfHb recorded during the Impella 5.5 run (and not elevated prior to device placement) was included (*n* = 123). From a practical standpoint, we were challenged by the fact that the bottom-most range of lab values for PfHb was “<30,” and therefore we do not have the actual value for a significant number of patients but know that the value is < 30. Each patient’s highest recorded PfHb while on Impella 5.5 (Fig. 1) was used to categorize the cohort into top (high hemolysis [HH], *n* = 26) and bottom (low hemolysis [LH], *n* = 25) quintiles (Fig. 2).

Analysis between groups was performed to identify factors associated with hemolysis. The HH group was then analyzed for factors associated with death versus survival. Outcomes of interest included days on Impella 5.5, blood transfusion, stroke, vascular complication (defined as operative vascular intervention), new renal replacement therapy (RRT), intensive care unit (ICU) days, admission outcomes, and 30-day mortality.

### Statistical analysis

Categorical variables are summarized as count (percent) and compared using Chi-square or Fisher’s exact tests. Numerical variables are summarized as median (interquartile range) and compared using Wilcoxon rank-sum tests. Multivariable logistic regression identified risk factors for hemolysis. The Kaplan-Meier method was used to estimate survival. Statistical significance was prespecified at alpha level < 0.05. Analysis was performed in R version 4.2.3 (R studio version 1.1.456).

## Results

### Preoperative characteristics

Baseline characteristics of included patients are summarized in Table 1. The cohort was 82.4% (42/51) male and the median age was 61.0 (50.0–67.0). There was no significant difference in etiology of heart failure between HH and LH patients (*p* = 1.00). HH patients presented with significantly higher admission SCAI stages (*p* = 0.008). Otherwise, there was no significant difference in preoperative risk factors between groups. Six patients (11.8%) had an Impella 5.5 placed at an outside hospital prior to transfer to our center.

**Table 1:** Preoperative and postoperative comparison of high hemolysis and low hemolysis cohorts

	Total (*N* = 51)	LH Group (Bottom 20%, *N* = 25)	HH Group (Top 20%, *N* = 26)	*p* value
**Patient Characteristics**				
Age (median [IQR])	61.00 [50.00, 67.00]	64.00 [49.00, 69.00]	58.50 [50.50, 63.75]	0.503
Gender (%)				0.948
Female	9 (17.6)	5 (20.0)	4 (15.4)	
Male	42 (82.4)	20 (80.0)	22 (84.6)	
Body Surface Area (median [IQR])	1.95 [1.83, 2.09]	1.95 [1.87, 2.05]	1.96 [1.81, 2.17]	0.873
History of smoking (%)	25 (49.0)	14 (56.0)	11 (42.3)	0.485
Etiology of Heart Failure (%)				1.000
Ischemic	28 (54.9)	14 (56.0)	14 (53.8)	
Non-ischemic	23 (45.1)	11 (44.0)	12 (46.2)	
Impella 5.5 placed at OSH (%)	6 (11.8)	2 (8.0)	4 (15.4)	0.701
**Admission SCAI Stage (%)**				**0.008**
**A**	**3 (5.9)**	**0 (0.0)**	**3 (11.5)**	
**B**	**6 (11.8)**	**4 (16.0)**	**2 (7.7)**	
**C**	**20 (39.2)**	**13 (52.0)**	**7 (26.9)**	
**D**	**14 (27.5)**	**8 (32.0)**	**6 (23.1)**	
**E**	**8 (15.7)**	**0 (0.0)**	**8 (30.8)**	
Preoperative RRT (%)	15 (29.4)	4 (16.0)	11 (42.3)	0.079
Preoperative ejection fraction (%) (median [IQR])	20.00 [15.00, 30.00]	20.00 [15.00, 27.75]	22.50 [15.00, 30.00]	0.353
**Final preoperative lactate (median [IQR])**	**1.20 [1.00**,** 2.00]**	**1.10 [0.85**,** 1.45]**	**1.65 [1.12**,** 2.40]**	**0.025**
Final preoperative hemodynamics (median [IQR])				
Mean arterial pressure	73.00 [65.00, 78.00]	74.00 [69.00, 76.00]	70.00 [64.00, 82.00]	0.750
Central venous pressure	9.00 [5.00, 11.00]	7.00 [5.00, 11.00]	9.00 [6.00, 14.25]	0.325
Cardiac output	4.23 [3.76, 5.77]	4.16 [3.70, 5.92]	4.27 [3.80, 5.27]	0.792
Impella 5.5 Indication				0.228
Acute MI	15 (29.4)	8 (32.0)	7 (26.9)	
CHF Exacerbation	13 (25.5)	6 (24.0)	7 (26.9)	
Post-Cardiotomy	10 (19.6)	5 (20.0)	5 (19.2)	
Prior Impella Complication	9 (17.6)	2 (8.0)	7 (26.9)	
Arrhythmia	2 (3.9)	2 (8.0)	0 (0.0)	
Postpartum	2 (3.9)	2 (8.0)	0 (0.0)	
**Postoperative Factors**,** Complications**,** and Outcomes**				
Impella Site of Placement (%)				0.079
Aortic graft	3 (5.9)	3 (12.0)	0 (0.0)	
Left axillary	2 (3.9)	0 (0.0)	2 (7.7)	
Right axillary	46 (90.2)	22 (88.0)	24 (92.3)	
**Total Impella 5.5 days (median [IQR])**	**14.00 [6.50**,** 25.00]**	**10.00 [6.00**,** 15.00]**	**23.50 [9.00**,** 41.00]**	**0.001**
**Additional MCS with Impella 5.5 (%)**	**22 (43.1)**	**6 (24.0)**	**16 (61.5)**	**0.015**
**Additional MCS Days (median [IQR])**	**0.00 [0.00**,** 6.50]**	**0.00 [0.00**,** 2.00]**	**4.00 [0.00**,** 15.25]**	**0.005**
**Transfusions on Impella 5.5 (median [IQR])**				
**Packed red blood cells**	**8.00 [3.00**,** 17.00]**	**4.00 [2.00**,** 9.00]**	**12.50 [6.50**,** 28.00]**	**0.001**
**Fresh Frozen Plasma**	**0.00 [0.00**,** 5.00]**	**0.00 [0.00**,** 2.00]**	**2.50 [0.00**,** 7.00]**	**0.033**
**Platelets**	**1.00 [0.00**,** 4.50]**	**0.00 [0.00**,** 2.00]**	**3.00 [1.00**,** 6.75]**	**0.002**
Complications (%)				
New RRT	12 (23.5)	6 (24.0)	6 (23.1)	1.000
Operative vascular complication	4 (7.8)	2 (8.0)	2 (7.7)	1.000
Stroke	7 (13.7)	4 (16.0)	3 (11.5)	0.955
ICU days (median [IQR])	26.00 [15.00, 40.50]	25.00 [14.00, 34.00]	27.50 [16.50, 61.50]	0.270
LOS (median [IQR])	32.00 [21.00, 53.00]	41.00 [22.00, 49.00]	27.50 [20.25, 62.25]	0.917
Days survived after Impella 5.5 placed (median [IQR])	37.00 [12.50, 333.50]	47.00 [12.00, 416.00]	30.50 [14.50, 82.75]	0.509
30-day mortality (%)	24 (47.1)	11 (44.0)	13 (50.0)	0.882
Survived admission (%)	21 (41.2)	14 (56.0)	7 (26.9)	0.068
Survival outcome (%)				0.253
Durable LVAD	2 (3.9)	2 (14.3)	0 (0.0)	
Transplanted	10 (19.6)	5 (35.7)	5 (71.4)	
Recovered	9 (17.6)	7 (50.0)	2 (28.6)	

Abbreviations: High Hemolysis (HH), Low Hemolysis (LH), Outside Hospital (OSH), Renal Replacement Therapy (RRT) Society for Cardiovascular Angiography & Interventions (SCAI), Mechanical Circulatory Support (MCS), Intensive Care Unit (ICU), Length of Stay (LOS), Left Ventricular Assist Device (LVAD)

### Postoperative factors and complications

The majority of patients in both groups had the device placed in the right axillary artery (LH [84.0% (21/25)] versus HH [92.3% (24/26)], *p* = 0.103). HH patients had significantly more Impella 5.5 days (23.5 versus 10.0, *p* = 0.001) and significantly more additional MCS (defined as concurrent extracorporeal membrane oxygenation or percutaneous right ventricular assist device) in place (16/26 [61.5%] versus 6/25 [24.0%], *p* = 0.015). The six LH patients who required additional MCS were on VA ECMO. The breakdown of additional MCS for HH patients is as follows: VA ECMO (5/26 [19.2%]), percutaneous OxyRVAD (6/26 [23.1%]), VA ECMO converted to OxyRVAD (3/26 [11.5%]), and Impella RP (2/26 [7.7%]). Furthermore, the HH cohort spent significantly more days on additional MCS (4.00 [0.00, 15.25]) than the LH cohort (0.00 [ 0.00, 2.00]) (*p* = 0.005). HH patients additionally had significantly more transfusions of packed RBCs (12.5 versus 4.0, *p* = 0.001), fresh frozen plasma (2.5 versus 0.0, *p* = 0.033), and platelets (3.0 versus 0.0, *p* = 0.002). There was no significant difference between groups regarding new RRT, operative vascular complications, or strokes. Because incidence of death was high and because many patients did not require any RRT, the number to report for renal recovery (including those on preoperative RRT and those newly on postoperative RRT) is low. The renal recovery rate (if applicable) in each group was 12.5% (1/8) in the LH cohort and 11.1% (2/18) in the HH cohort. Multivariable logistic regression identified the presence of additional MCS (OR 10.82, *p* = 0.004) and increased Impella days (OR 1.13 *p* = 0.006) as risk factors for hemolysis.

### Outcomes and survival

There was no statistically significant difference between HH and LH duration (days) of survival after Impella 5.5 placement (30.5 [14.5, 82.8] and 47 [12, 416] respectively) or 30-day survival (42.3% [11/26] and 44.0% [11/25] respectively). With regard to cardiac outcomes, 11/25 (44.0%) LH patients and 19/26 (73.1%) HH patients died, two (8.0%) LH patients transitioned to durable VAD, five patients were transplanted from each cohort (LH [20.0%] versus HH [19.2%]), and 7/25 (28.0%) LH patients recovered. There was no statistically significant difference between cohorts across these outcomes (*p* = 0.075). More LH patients (14/25 [56.0%]) than HH patients (7/26 [26.9%]) survived the admission, showing a trend toward significance (*p* = 0.068).

**Fig. 3:**
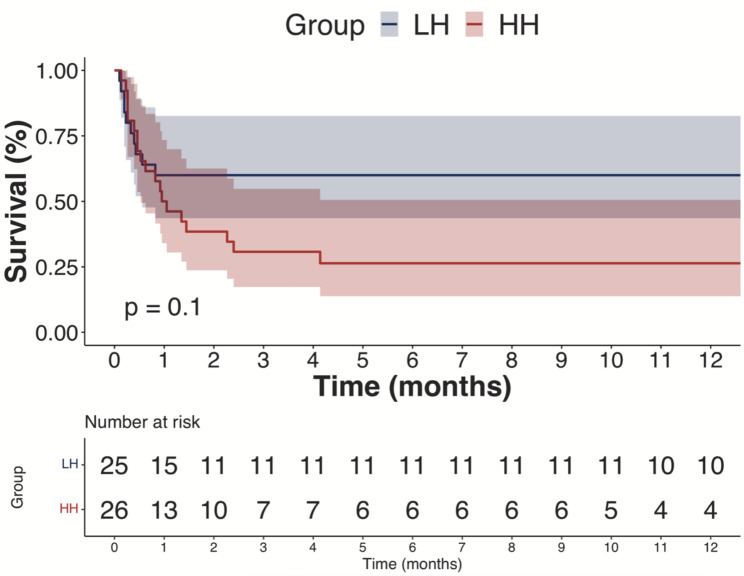
Kaplan-Meier Survival of high hemolysis (HH) and low hemolysis (LH) cohorts

The Kaplan-Meier survival estimate (95% CI) at 1 month for HH versus LH groups was 50.0% (34.0 − 73.4%) and 60.0% (43.6 -82.6%) respectively, and 26.4% (13.8 − 50.6%) and 60.0% (43.6 -82.6%) respectively at 6 months (*p* = 0.1, Fig. 3).

### High hemolysis sub-analysis

Finally, the HH group was analyzed for factors associated with death versus survival (Table 2). Median Impella 5.5 days to peak PfHb in this cohort was 8.5 (1.25, 19.25) with no significant difference between survivors and those who died. HH group survivors were found to have significantly fewer platelet transfusions (2.0 vs. 5.0, *p* = 0.01), lower bilirubin (1.20 vs. 5.40, *p* = 0.003) and less days of PfHb elevation (defined as PfHb > 30, 3.0 v 6.0, *p* = 0.007), compared to those who died.

**Table 2:** Analysis of the top quintile (HH group) for factors associated with survival versus death

Analysis of Top Quintile (HH patients)	Total (*N* = 26)	Died (*N* = 19)	Survived (*N* = 7)	*p* value
Total Impella 5.5 days (median [IQR])	23.50 [9.00, 41.00]	21.00 [8.00, 27.50]	44.00 [30.50, 60.50]	0.064
Impella 5.5 days to peak PfHb (median [IQR])	8.50 [1.25, 19.25]	9.00 [2.00, 20.00]	7.00 [1.00, 10.50]	0.323
Transfusions on Impella 5.5 (median [IQR])				
Packed red blood cells	12.50 [6.50, 28.00]	18.00 [7.00, 29.00]	8.00 [6.00, 9.50]	0.183
Fresh Frozen Plasma	2.50 [0.00, 7.00]	3.00 [0.00, 9.50]	1.00 [0.00, 3.50]	0.271
**Platelets**	**3.00 [1.00**,** 6.75]**	**5.00 [2.50**,** 9.00]**	**1.00 [0.50**,** 1.50]**	**0.010**
Labs at time of highest PfHb (median [IQR])				
PTT	43.00 [38.85, 48.75]	43.90 [38.10, 47.20]	42.10 [41.60, 52.55]	0.470
LDH	1073.50 [706.25, 1941.50]	1182.00 [978.50, 2196.50]	719.00 [516.50, 1080.50]	0.060
**Bilirubin**,** Total**	**3.80 [1.65**,** 8.20]**	**5.40 [3.15**,** 11.60]**	**1.20 [0.85**,** 2.45]**	**0.003**
**Days of elevated PfHb (median [IQR])**	**6.00 [3.00**,** 7.75]**	**6.00 [5.00**,** 8.00]**	**3.00 [1.00**,** 4.50]**	**0.007**

Abbreviations: High Hemolysis (HH), Plasma Free Hemoglobin (PfHb), Partial thromboplastin time (PTT), Lactate dehydrogenase (LDH)

## Discussion

We aimed to better characterize the complex complication of hemolysis in a real-world series of patients supported with the Impella 5.5 percutaneous LVAD, which is unprecedented.

To provide insight into MCS and hemolysis management at our center, it is our practice to determine ECMO and Impella flows based on body surface area for a cardiac index of 2.2–2.4, unless a lower level of support is clinically indicated. In someone with suspected hemolysis, there was a step-wise protocol for troubleshooting. Device position was checked using radiography and echocardiogram (TTE or TEE if necessary), and would be promptly adjusted if found to be malpositioned, resulting in an improvement of symptoms. Additionally, purge pressures were checked for any issues. There was no standardized anticoagulation protocol for non-complicated patients, but in cases of continued hemolysis, as long as the patient had no clinical contraindications, the level of anticoagulation was increased either by increasing systemic heparin drip or adding heparin to the bicarbonate purge solution. Finally, in those patients who were judged to be able to tolerate a lower level of hemodynamic support, the revolutions per minute on the device were lowered. Ultimately, several Impella supported patients needed rescue therapy with ECMO if the above measures did not help.

Study limitations include its retrospective nature, relatively low sample size of both cohorts, and the lack of a consistent and well-established hemolysis definition in MCS. Because of this lack of a hemolysis definition, we are unable to prove with evidence that hemolysis is an independent risk factor for adverse events. Furthermore, and related to the retrospective nature of the study, this analysis is hampered by the fact that no consistent clinical protocol for sending hemolysis labs on patients in the postoperative period throughout the entirety of the study period was in place. This, in turn, affected the sample size as 39 patients of the original 169 had to be excluded as there was no PfHb drawn.

Further studies involving larger sample sizes, longer follow-up periods, and a protocolized manner of PfHb sampling will be necessary to further elucidate factors contributing to percutaneous LVAD-associated hemolysis and its sequelae.

## Conclusions

The authors appreciate that hemolysis is an indicator of an increased risk of poor outcomes, and as such should be aggressively tracked and minimized whenever possible using the approaches outlined above. The HH patients spent more days on Impella 5.5, were on additional MCS, and required more transfusions. HH patients who survived required fewer platelet transfusions, had lower bilirubin, and had less days of elevated PfHb.

Hemolysis in the field of MCS has historically been variably defined, and this assessment of a series of patients with a contemporary MCS device may add further insight into the characteristics of patients experiencing clinically significant hemolysis in the field.

## Electronic supplementary material

Below is the link to the electronic supplementary material.

Supplementary Material 1

## Data Availability

No datasets were generated or analysed during the current study.

## Abbreviations

FDA: U.S. Food & Drug Administration
HH: High Hemolysis
ICU: Intensive Care Unit
INTERMACS: Interagency Registry for Mechanically Assisted Circulatory Support
LDH: Lactate Dehydrogenase
LH: Low Hemolysis
LVAD: Left Ventricular Assist Device
MCS: Mechanical Circulatory Support
MIH: Modified Index of Hemolysis
PfHb: Plasma Free Hemoglobin
RBCs: Red Blood Cells
RRT: Renal Replacement Therapy
SCAI: Society for Cardiovascular Angiography and Interventions
