# Supplementary material for: Evaluation of hemolysis in patients supported with Impella 5.5: a single center experience
Source: J Cardiothorac Surg. 2025 Mar 1;20:143. doi: 10.1186/s13019-025-03352-7 (PMC11871597; doi:10.1186/s13019-025-03352-7)
Supplement: Supplementary file 1 — Supplementary Material 1 [file 13019_2025_3352_MOESM1_ESM.docx]

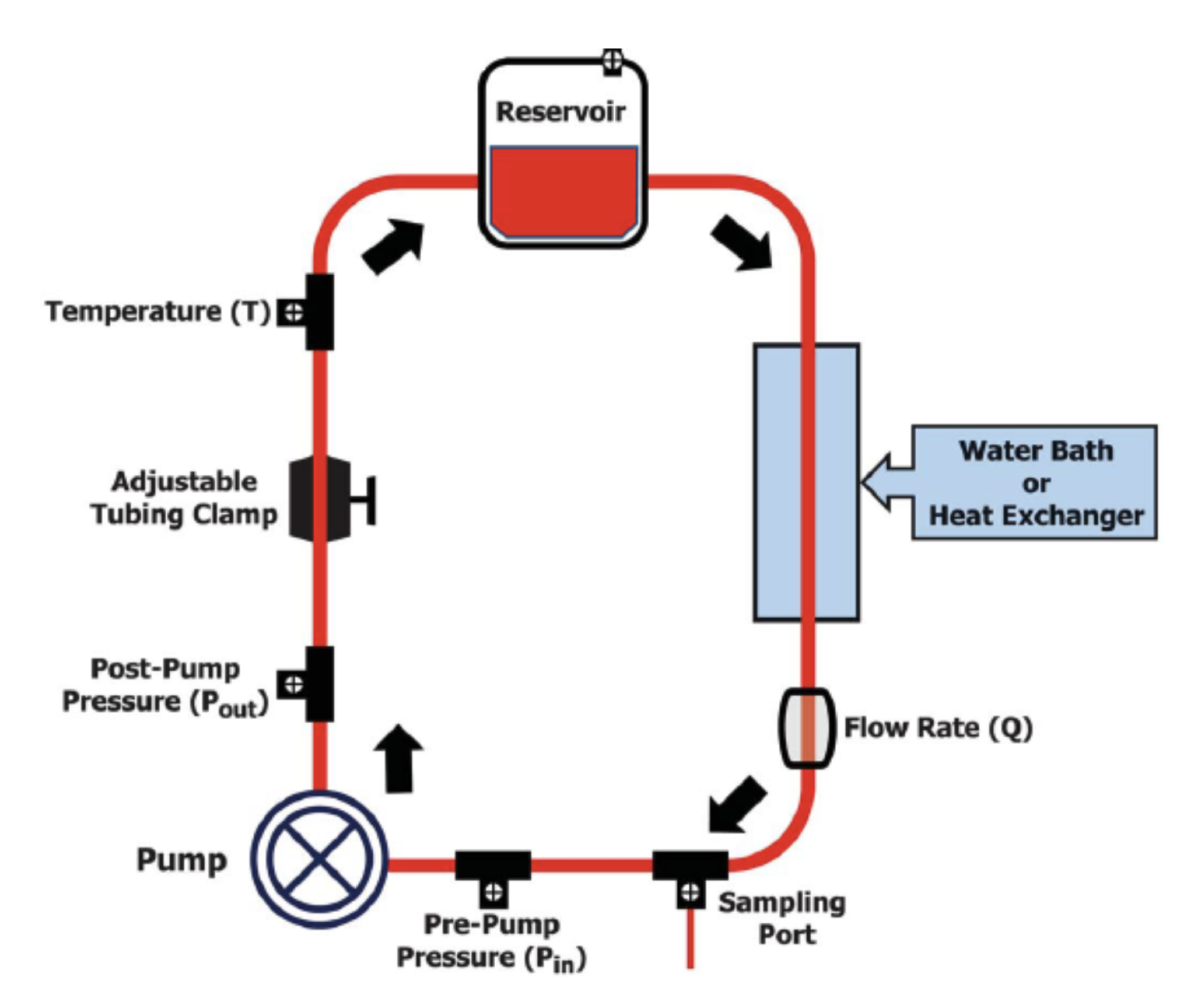


Figure S1. Typical hemolysis testing model for FDA approval of implantable pumps^14^ (Image: Malinauskas R, Rinaldi J, Jamiolkowski M, Lu Q. In Vitro Dynamic Hemolysis Testing of Blood Pumps: Updating the ASTM F1841 Testing Standard. 2021 FDA Science Forum: fda.gov; 2021.)


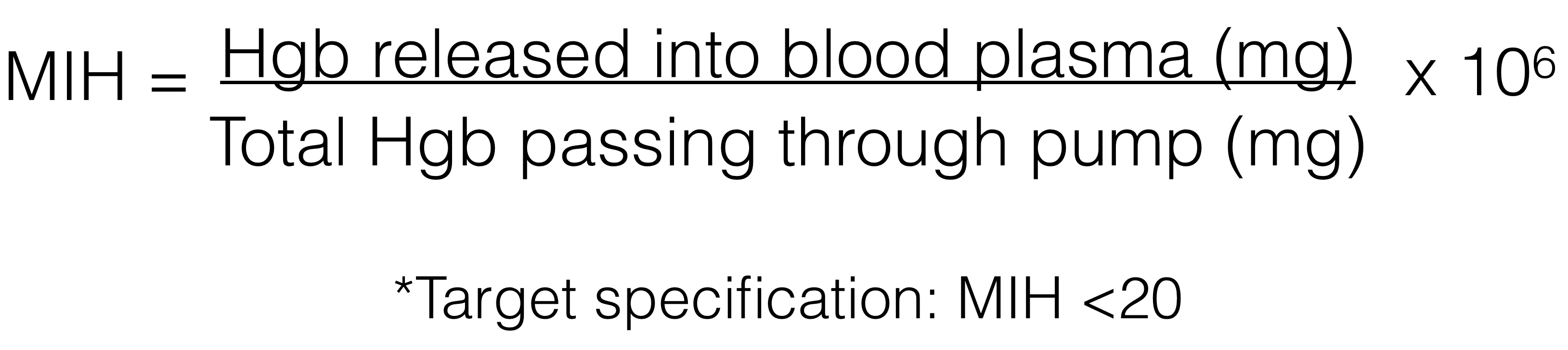


Figure S2. Equation for Modified Index of Hemolysis (MIH), which represents rate of blood damage over time in FDA recognized hemolysis testing
